# Coronavirus Disease (COVID-19) and Pediatric Patients: A Review of Epidemiology, Symptomatology, Laboratory and Imaging Results to Guide the Development of a Management Algorithm

**DOI:** 10.7759/cureus.7485

**Published:** 2020-03-31

**Authors:** Ali Hasan, Noormah Mehmood, Jamie Fergie

**Affiliations:** 1 Pediatrics, Driscoll Children's Hospital, Corpus Christi, USA; 2 Pediatric Infectious Diseases, Driscoll Children's Hospital, Corpus Christi, USA

**Keywords:** coronavirus disease, covid-19

## Abstract

Coronavirus disease (COVID-19) has been declared a worldwide pandemic. Compared to adults, there has been a significantly smaller number of reported cases of COVID-19 in the pediatric population, although the incidence is increasing every day. This article looks to review specific epidemiological factors, symptomatology, laboratory and imaging workup, and other relevant metrics derived from the limited published literature that are specific to the pediatric population, to provide a review for the pediatric practitioner and guide, in part, the creation of a clinical algorithm for the management of COVID-19 in the pediatric population that can be utilized by pediatric institutions.

## Introduction and background

The novel coronavirus disease 2019 (COVID-19), caused by severe acute respiratory syndrome coronavirus two (SARS-CoV 2), is a member of *Betacoronaviruses*, like the former human coronaviruses SARS coronavirus (SARS-Cov) and the Middle East respiratory syndrome (MERS) [1]. Human coronaviruses are positive-sense, long (30,000 base pairs) single-stranded RNA viruses [2-3]. COVID-19 was first detected in humans towards the end of 2019 and the first cases were traced back to Wuhan city (Hubei province) in China [1]. The virus appears to spread via human to human transmission in a similar fashion to influenza and several viruses causing upper respiratory infections, i.e., through contact with secretions from infected individuals [1]. There is also concern regarding airborne transmission as well as oro-fecal transmission. The virus predominantly replicates in the respiratory system during the prodromal period, which further contributes to the transmission of the disease as patients may still be harboring the infection in the absence of symptoms [4]. After the initial reports of infection, in the following several weeks, COVID-19 outbreaks were reported in South Korea, Iran, and Italy. This was quickly followed by several other European, Asian, and North and South American countries reporting cases. COVID-19 was declared a pandemic by the World Health Organization (WHO) on March 11, 2020.

Compared to adults, there have been significantly less reported cases of COVID-19 in the pediatric population [5]. As of February 2020, 2.4% of the 75,465 cases (confirmed and suspected) in China were reported in the pediatric population [5]. This article looks to review specific epidemiological factors, symptomatology, laboratory, and imaging workup and other relevant metrics derived from the limited published literature that are specific to the pediatric population to provide a review for the pediatric practitioner and guide in part towards the creation of an interim algorithm for the management of COVID-19 in the pediatric population.

## Review

Epidemiology

Reviewing published literature, Jiehao et al., in their case series of 10 children with the 2019 novel coronavirus, reported that the age group of patients affected was between three and 131 months with a mean age of 74 months with a male to female ratio of 1:1.5 [5].

Xia et al. noted 65% of the affected patients to be male within their subset of 20 pediatric inpatients with COVID-19 infection [6]. The age range within this group of affected patients was one day to 14 years with a median age of two years [6]. Seventy percent of the affected patients within this subset were under the age of three years [6]. One of the patients had a history of epilepsy as a sequela of previous viral encephalitis and two patients had a history of atrial septal defect (ASD) repair surgery [6]. The authors noted five further patients with a history of congenital or acquired diseases (unspecified within the reported study), which the authors purported to indicate that children with underlying diseases would have a greater susceptibility to COVID-19 [6]. Jiehao et al. noted within their study that the mean incubation period in their set of pediatric patients from household exposure to a symptomatic adult case was six and a half days, which they noted to be suggestive of a longer incubation period than what is being reported in adults [5].

Dong et al., in their pre-publication release data looking at the epidemiology of COVID-19 among children in China, reviewed 2143 cases of which 731 were laboratory confirmed and 1412 were suspected cases. They found the median age among these cases to be seven years with 56.6% of the cases being boys [7].

Overall, the epidemiological data suggests a slightly higher percentage of affected cases to be male. The age range of affected patients is wide, with concern regarding a higher propensity of illness in patients with pre-existing diseases. This may represent either worse symptoms resulting in a higher rate of testing or may indicate an increased susceptibility to illness with underlying disease.

Symptomatology

Xia et al. noted in their study of pediatric COVID-19 cases that eight (80%) patients had a fever, six (60%) had a cough, four (40%) had a sore throat, three (30%) had a stuffy nose, and two (20%) had sneezing and rhinorrhea. None of the patients had diarrhea or dyspnea during the course of their illness [5].

Xia et al. report the presence of fever, which was defined as axillary temperature over 37.3°C in 12 cases (12/20, 60%), cough in 13 cases (13/20, 65%), diarrhea in three cases (3/20, 15%), nasal discharge in three cases (3/20, 15%), sore throat in one case (1/20, 5%), vomiting in two cases (2/20, 10%), tachypnea in two cases (2/20, 10%), and fatigue in one case (1/20, 5%) [6]. They also further noted physical exam findings when assessed by medical personnel to be rales in three cases (3/20, 15%), retraction signs in one case (1/20, 5%), and cyanosis in one case (1/20, 5%) [6].

Dong et al. characterized, in looking at their data of 2143 pediatric patients with laboratory diagnosed and/or clinically suspicious cases of COVID-19 infection, the severity of illness as asymptomatic, mild (predominantly upper respiratory tract infectious symptoms with no frank respiratory distress), moderate (presence of pneumonia, frequent fever and cough but with no obvious hypoxemia), severe (presence of dyspnea with central cyanosis, oxygen saturation <92% with other hypoxia manifestations) and critical (acute respiratory distress syndrome (ARDS), respiratory failure, shock, encephalopathy, myocardial injury, heart failure, coagulation dysfunction, and organ dysfunction) [7]. With these clinical parameters, they found 4.4% of cases to be asymptomatic, 50.9% of cases to be mild, and 38.8% of the cases to be in the moderate range accounting for 94.1% of all cases [7]. They also noted the proportion of severe and critical cases to be inversely proportional to the age range, with the age group of less than one year old having 10.6% of the severe and/or critical cases [7].

Imaging

Chest radiographs revealed a unilateral patchy infiltrate in four (40%) of 10 patients with COVID-19 reported by Jiehao et al. [5]. Xia et al. further looked to examine the chest CT findings at various stages of the COVID 19 process. At the early stage of the disease, they noted six patients presented with unilateral pulmonary lesions (6/20, 30%), 10 with bilateral pulmonary lesions (10/20, 50%), and one pediatric patient and three neonates had no abnormalities on chest CT (4/20, 20%) [6]. Sub-pleural lesions with localized inflammatory infiltration were found in all children [6]. Ten patients (10/20, 50%) were noted to have “halo sign” consolidation, 12 patients (12/20, 60%) had ground-glass opacities, four patients (4/20, 20%) had “fine mesh shadows,” and tiny nodules were detected in three patients (3/20, 15%) [6]. No patients were noted to have signs of pleural effusion and lymphadenopathy on CT scan [6].

Labs

Jiehao et al. noted within their laboratory findings: median white blood cell count (WBC) 7.35×109/L, C-reactive protein (CRP) 7.5 mg/L, procalcitonin (PCT) 0.07 ng/dL, creatine kinase-myocardial band (CK-MB) 23 U/L, alanine aminotransferase (ALT) 18.5 U/L, aspartate aminotransferase (AST) 27.7 U/L, urea 3.1 mmol/L, creatinine 35.5 μmol/L, lactate dehydrogenase (LDH) 25 U/L, and D-dimer 0.45 μg/mL; influenza virus A and B were negative [5]. The study also showed that all patients had 2019-nCoV RNA detected in nasopharyngeal and throat swabs within four to 48 hours after the onset of symptoms and 2019-nCoV RNA in nasopharyngeal or throat swabs was no longer detectable within six to 22 days (with a mean of 12 days) after the onset of illness. Six of these patients had fecal samples tested, and 5 (83.3%) were positive for 2019-nCoV RNA. The authors also noted with concern that the five patients still had 2019-nCoV RNA detected in feces within 18-30 days after illness onset at the time of publication of their findings. Five patients also had serum and urine samples tested and were negative for 2019-nCoV RNA.

For Xia et al. (per their reference ranges used), WBC was normal (5.5-12.2) in 14 cases (14/20, 70%), decreased (<5.5) in four cases (4/20, 20%), and increased (>12.2) in two cases (2/20, 10%); ALT increased (>40 IU/L) in five cases (5/20, 25%),CK‐MB increased in 15 cases (15/20, 75%), and PCT (>0.05) increased in 16 cases (16/20, 80%). Eight patients were co-infected with other pathogens (8/20, 40%), including influenza viruses A and B, mycoplasma, respiratory syncytial virus (RSV), and cytomegalovirus (CMV) [6]. Further, four cases had abnormal electrocardiogram (EKG) events, including atrial arrhythmia, first-degree atrioventricular (AV) block, atrial and ventricular premature beats, and incomplete right bundle branch block [6].

Elevations in CK-MB and EKG changes are of particular concern, as they may be indicative of myocarditis as a potential complication of COVID-19. Lippi et al., in their electronic data review (which was not specific for pediatric patients), also noted the cardiac Troponin I value significantly increased in patients with severe COVID-19 infection [8].

Transmission

Jiehao et al. noted the mean number of secondary symptomatic cases in the household exposure setting was 2.43, which is indicative of the basic reproductive number for pediatric COVID-19 cases and proof of direct transmission [5]. Xia et al. noted within their subset of 20 cases that 13 pediatric patients (13/20, 65%) had an identified history of close contact with COVID‐19 diagnosed family members, again supporting proof of direct transmission [6].

A particular source of concern is the paucity of data on the vertical transmission potential of COVID-19 pneumonia in pregnant women [9]. Chen et al. retrospectively reviewed the medical records for nine pregnant women with laboratory-confirmed COVID-19. The evidence of intrauterine vertical transmission through testing for the presence of SARS-CoV-2 in amniotic fluid, cord blood, and neonatal throat swab samples was assessed. Breastmilk samples were also collected and tested from patients after the first lactation [9].

They reported that within this subset of patients nine live-births delivered via cesarian section were recorded. No neonatal asphyxia was observed in the newborn babies. All nine newborns had a one-minute Apgar score of eight to nine and a five-minute Apgar score of nine to 10. Six patient’s amniotic fluid, cord blood, neonatal throat swab, and mother’s breastmilk samples were tested for SARS-CoV-2, and all samples tested negative for the virus [9]. They noted within this subset of patients that the clinical characteristics of the disease were similar in pregnant and non-pregnant adults and that they did not note any evidence of intrauterine infection caused by vertical transmission in women who develop COVID-19 pneumonia in late pregnancy [9].

Management algorithm

**Figure 1:**
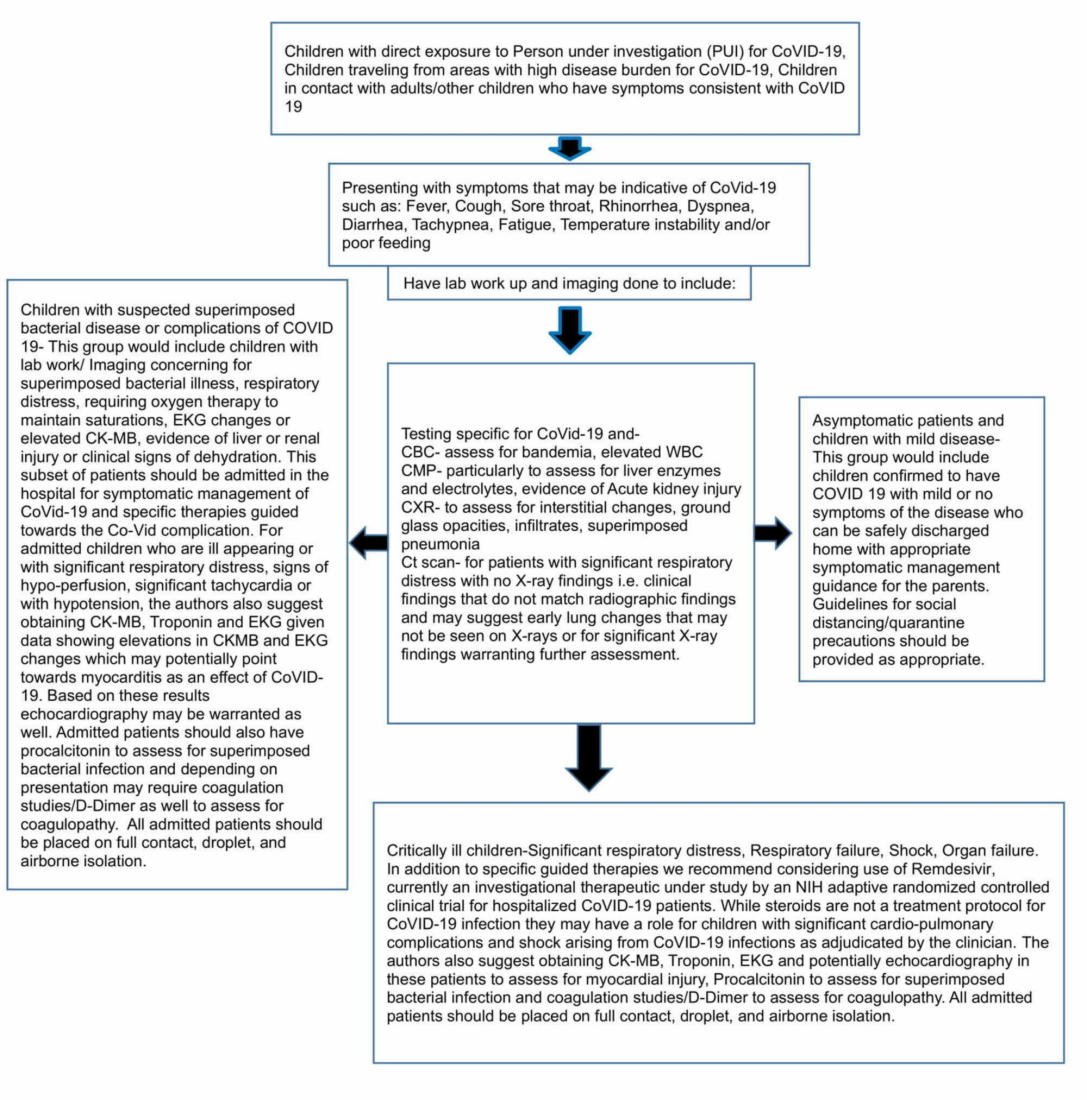
Proposed management algorithm and clinical pathway for pediatric patients with suspected COVID-19 infection COVID-19: coronavirus disease 2019; CBC: complete blood count; CMP: complete metabolic panel; CXR: chest X-ray; CT scan: computed tomography scan; CK-MB: creatine kinase myocardial band

At the time of publication of this article, the U.S. Food and Drug Administration (FDA) currently has no approved medications to treat patients with COVID-19. As such, management algorithms, particularly for the pediatric patient, are based at least in part on clinical opinion. Partially based on the data reviewed above and partially upon the opinion of the authors of the article, looking specifically at pediatric COVID-19, we recommend the investigation and management pathway as displayed in Figure 1.

## Conclusions

COVID-19 related pediatric disease has an array of symptomatic presentations and outcomes. While the majority of those on the spectrum of the disease will recover well with symptomatic care, this article serves to highlight how myocardial disease, lung pathology, and the substantially higher risk of mortality in certain sub-populations should be kept in the mind. As such, based on our limited data, the authors favor an approach that relies on ensuring potential markers of poorer outcomes, such as evidence of organ dysfunction, evidence of superimposed bacterial infection, and other metrics highlighted in the article, are screened for at an earlier stage. For those children that are deemed sick enough to require admission, the potential need for further investigation for myocardial disease, coagulopathy, and organ damage should be kept in mind.
